# Structural changes in the Russian health care system: do they match European trends?

**DOI:** 10.1186/s13561-022-00373-z

**Published:** 2022-05-26

**Authors:** Sergey Shishkin, Igor Sheiman, Vasily Vlassov, Elena Potapchik, Svetlana Sazhina

**Affiliations:** 1 Centre for Health Policy, National Research University Higher School of Economics, 4 Slavyanskaya Ploshchad, Building 2, Moscow, Russia 109074; 2 Department of Health Care Administration and Economics, School of Politics and Governance, Faculty of Social Sciences, National Research University Higher School of Economics, 20 Myasnitskaya Ulitsa, Moscow, Russia 101000

**Keywords:** Health care system, Health service provision, Primary care, Inpatient care, Structural changes, Semashko model

## Abstract

**Вackground:**

In the last two decades, health care systems (HCS) in the European countries have faced global challenges and have undergone structural changes with the focus on early disease prevention, strengthening primary care, changing the role of hospitals, etc. Russia has inherited the Semashko model from the USSR with dominance of inpatient care, and has been looking for the ways to improve the structure of service delivery. This paper compares the complex of structural changes in the Russian and the European HCS.

**Methods:**

We address major developments in four main areas of medical care delivery: preventive activities, primary care, inpatient care, long-term care. Our focus is on the changes in the organizational structure and activities of health care providers, and in their interaction to improve service delivery. To describe the ongoing changes, we use both qualitative characteristics and quantitative indicators. We extracted the relevant data from the national and international databases and reports and calculated secondary estimates. We also used data from our survey of physicians and interviews with top managers in medical care system.

**Results:**

The main trends of structural changes in Russia HCS are similar to the changes in most EU countries. The prevention and the early detection of diseases have developed intensively. The reduction in hospital bed capacity and inpatient care utilization has been accompanied by a decrease in the average length of hospital stay. Russia has followed the European trend of service delivery concentration in hospital-physician complexes, while the increase in the average size of hospitals is even more substantial. However, distinctions in health care delivery organization in Russia are still significant. Changes in primary care are much less pronounced, the system remains hospital centered. Russia lags behind the European leaders in terms of horizontal ties between providers. The reasons for inadequate structural changes are rooted in the governance of service delivery.

**Conclusion:**

The structural transformations must be intensified with the focus on strengthening primary care, further integration of care, and development of new organizational structures that mitigate the dependence on inpatient care.

## Background

In the last two decades, health care systems in the European Union countries have faced global challenges, including aging populations, a substantial rise in chronic and multiple diseases, the emergence of new medical and information technologies, and a growing citizen awareness of the role of a healthy lifestyle in disease prevention [1]. The responses of health systems to these challenges included structural changes in their organization with a focus on the promotion of healthy lifestyles and disease prevention, the growing scale of screening for early disease detection, strengthening primary care, changing the role of the hospitals, the development of chronic disease management programs, etc. [2, 3]

Studies of these trends address mostly Western countries. Much less attention has been paid to the post-Soviet countries. In this paper, we study structural changes in the health care in Russia. Russian health care has inherited the Semashko model of health care organization. Its main distinction is state-centered financing, regulation, and provision of health care. The model has specific forms of provider organization, for example, outpatient clinics (polyclinics) with a large number of various specialists, the separation of care for adults and children, and large highly-specialized hospitals [4].

The Soviet and post-Soviet health systems have been underfunded. Public health funding in the 1990s dropped almost by one third in real terms [5]. The organization of medical care in the 1990s has not changed significantly relative to Soviet times, and the system has adapted through the reduction in the volume of services and increased payments by patients, frequently informal [6]. The surge in oil prices after 2000 allowed health funding to increase and while encouraged noticeable changes in service delivery.

The changes in the Russian health system have been discussed in the literature mostly focusing on specific sectors and health finance reforms [5, 7–17]. But these changes in different sectors were not analyzed together, from a single methodological position, as changes in the structural characteristics of the Russian health care system, i.e. the changes in the ratio of different types of medical care, in the structure of medical service providers, in functionalities and modes of their interaction.

The objective of this paper is to explore the entire complex of structural changes over the past two decades in comparison with European trends. What were the structural changes in European health care systems, what were they like in Russia, and how can their differences be explained?

## Methods

### Study design

We followed a six-step methodological framework. The first stage involved designation of the types of medical care and the types of structural changes for identification and comparison. We considered four main areas of medical care delivery: preventive activities, primary care, inpatient care, long-term care. We focused on three different dimensions of structural changes: i) changes in the organizational structure of medical service providers; ii) changes in the structure of their activities (in its types and in their coverage of the population / patients); iii) changes in the organization of interaction between different service providers.

The second stage consisted of identifying for each type of medical care the changes in these three dimensions in the last twenty years before the COVID-19 pandemic. We described the changes that met two criteria: 1) these changes are assessed in the OECD, WHO, and World Bank reviews, and other review publications on this topic as the most noteworthy characteristics of the development of European health care systems, and 2) they have spread in a large number of European countries.

The changes identified according to the formulated criteria cover not all dimensions of structural changes for each type of medical care. For preventive activities, there are changes in the types of activities and in their coverage of the population. In primary and inpatient care, there are changes in the organizational structure of service providers, in the structure of their activities, and in the organization of interaction with other providers. In long-term care, there are changes in the structure of developed activities and their coverage of the population.

To describe the ongoing changes, we use both qualitative characteristics and, if possible, quantitative indicators that highlight them to the greatest extent.

The third stage involved detection of structural changes in four main areas of medical care delivery in Russia. We used the results of our previous studies and conducted an additional search for data characterizing structural changes in health care, using new statistical data, evidence derived from our survey of physicians and interviews with top managers in medical care system.

On the fourth stage we compared the identified structural changes in European health care systems (HCS) with the changes taking place in Russian health care. We identified the presence or absence of similar types of structural changes and the differences between them. The fifth stage was the consideration of the driving forces of structural changes in the Russian health care system. The sixth stage included discussion of the reasons for the distinctions with European developments.

### Data sources

To identify the main structural changes in medical care delivery during last twenty years we searched the literature addressing both European HCS and Russia in the all aspects of changes of health care system indicators, better classified by MeSH term “health care reform”. We searched MEDLINE using the query: (russia OR europ* OR “european union” OR semashko) AND health care reform [mh] AND 2000:2021[dp]). All 788 findings were checked manually and 86 were relevant. We also used sources snowballed from these reports and the grey literature related to Russian health care, including those in limited circulation, unpublished documents, memorandums, and presentations from our personal collections covering more than twenty years.

We also used data from an online survey of 999 primary care physicians (further – survey) conducted by the authors in April–May 2019. The respondents representing 82 out of 85 regions of the Russian Federation were asked about implementation of the national prophylactic medical examination program. We also interviewed four leading specialists of the national Ministry of Health on the criteria for the inclusion of the components into the program.

To identify the driving forces of structural changes in the Russian health care system, we used materials from 10 interviews on the issues of implementing state health care programs that we conducted in 2019 with current and former top-managers in the federal government and in five regional governments. We also used the grey literature as well as published reports.

We used statistical data from the international databases of OECD [18], WHO [19], World Bank [20], as well as the Russian sources — the Federal State Statistics Service [21] and the Russian Research Instuitute of Health [22]. The data was analyzed for the period from 2000 to the latest date with available data for both EU member states and Russia. To ensure the comparability of the composition of countries in different years, the analysis of the dynamics of some indicators was limited to EU 19 members, i.e. excluding Cyprus, Greece, Croatia, Bulgaria, Luxemburg, Malta, Netherland, Poland, and Romania. The averages for EU 19 estimates are based on population size-weighted averages. If the studied publications and databases did not contain the necessary indicators, we made our own estimates.

Each section of the paper contains a brief description of the main trends in the European countries, and then provides a comparative analysis of the corresponding changes in Russian health care. The comparison is followed by a discussion of the driving forces and the limitations of structural changes in Russia compared to the main European trends. We limited our analysis to the pre – COVID-19 pandemic years.

## Results

### The development of preventive activities

#### European HCS

Most of them have implemented health check-ups, and population and opportunistic screenings for the early detection of diseases. These activities are viewed as a way to improve outcomes by ensuring that health services can focus on diagnosing and treating disease earlier [23]. The population covered by screenings is high and growing. In Germany 81% of population between 50 and 74 years in 2014 had been tested for colorectal cancer at least once, in Austria 78%, France 60%, Great Britain 48% [24].

The impact of these activities on health outcomes depends on the selection of preventive services, as well as on their implementation in specific national contexts. The selection of preventive services is increasingly based on research into their potential impact on mortality and other health indicators, as well as their cost effectiveness, with some services being declined because of their inadequate input into health gains [25]. It is particularly important that screenings are focused on socially disadvantaged groups with the highest probability of disease identification and the expected benefits of their management. Therefore, screening programs are based on the evaluation of local needs. Physicians have discretion in the choice of patients for screenings, depending on their importance for specific groups of the population, and individual risks and preferences.

It is increasingly common for a screening program to include follow-up management of any detected illnesses, with the implication that policy makers design such programs as a set of interrelated preventive and curative activities [26].

#### Russia

The original Semashko model and the current legislation prioritize preventive activities, while their implementation has been limited by the chronic underfunding of the health system. In the 2000s, the priority of prevention campaigns was revitalized in the form of a national prophylactic medical examination program (Prophylactic Program, called Dispanserization) that is a set of health check-ups and screenings. The major expectation from this Prophylactic Program is the same as in European HCS [27].

To supplement the analysis of the Prophylactic Program, we analyzed the evidence base for the components of the program and interviewed leading specialists of the federal Ministry of Health on the criteria for the inclusion of the components into the program. We found that some screenings were not evidence based and effect on the population health and/or health of participants is small [28]. The screening package of the dispanserization was expanded and reduced couple of times, but still number of ineffective screenings are included in the package (electrocardiography (ECG) screening of healthy subjects, prostate specific antigen (PSA) screening of middle age and adult men, urinalysis and routine blood tests, mammography from age 40 etc.).

Primary care physicians play a major role in conducting screenings and check-ups as well as subsequent interventions. There are also public health units responsible exclusively for these preventive activities in big polyclinics. Polled in 2019, primary care physicians responded that in 11% of polyclinics check-ups are carried out in these departments only, and in 24% of primary care organizations the check-ups are conducted by district physicians as well as by staff of these preventive units.

Under the current Prophylactic Program, people over 40 are supposed to have a set of check-ups annually; those 18–39 every three years. Most children go through physicals only. The official estimates of the coverage of the eligible population in the Prophylactic Program are around 100% [29], while service providers are less optimistic. According to the survey, more than half of the respondents reported that this share was less than 60%, while 17.4% reported less than 20% [27].

An important shortcoming of the Prophylactic Program design and implementation is the gap between its major objective and the capacity of primary care. The shortage of primary care physicians does not allow the target groups to be provided with all preventive services. Physicians have to distort the service to their registered population and to underprovide the follow-up care of detected cases. The lack of a systematic approach, less focus on local conditions, and the lack of a professional autonomy of providers are the major distinctions between Russian prevention campaigns and similar activities in Europe.

The Prophylactic Program is built on the presumption that preventive activities should include the follow-up management of any detected conditions. There is some evidence, however, that this is not taking place: according to our survey, a half of primary care physicians are unaware of the results of check-ups and screenings. The reported coverage and quality of the follow-up management of identified cases are low: a half of the respondents indicate that less than 60% of patients with identified diseases become objects of the follow-up disease management. Only 7.7% of respondents indicate that a set of disease management services corresponds to a pattern of dispensary surveillance issued by the federal Ministry of Health. The majority reports that these requirements are met only for some patients or are not met at all.

Disease management of newly identified chronic and multiple cases is focused on process rather than outcome indicators. The information on the latter is very fragmented. According to our survey, a decrease in the number of disability days of chronic patients is reported by only 14% of physicians. More than a half of respondents are unaware of the number of emergency care visits and hospital admissions of their chronic patients.

### Strengthening primary care

#### European HCS

There is a trend of multi-disciplinary primary care practices or networks development and promotion of teamwork and providers coordination in response to the growing complexity of patients. In Spain, France, and the UK it is increasingly common for large general practices to serve more than 20,000 people and provide a wider spectrum of services than in traditional solo and group practices. These emerging extended practices include pharmacists, mental care professionals, dieticians, and sometimes 2–3 specialists [30, 31]. The role of nurses is also expanding. Most advanced nurses independently see patients, provide immunizations, health promotion, routine checks for chronically ill patients in all EU member states [32]. Related to these extended practices is the growing concentration of primary care providers via mergers and reconfigurations that increase the size of the units. The major benefits are economies of scale and scope through staff sharing and better integrated care.

There is also a general trend to strengthen the links with the local community, social care and hospitals [32]. Primary care providers are increasingly involved in chronic disease management programs together with other professionals in and out of general practices. Links with hospitals are developing beyond simple referral systems [33].

#### Russia

The trend of multidisciplinary practices development has greatly affected Russian health care. However, this trend in Russia differs significantly from the European HCS. It began in the 1980s, when large numbers of specialists were employed by polyclinics, which are the major providers of both primary care and outpatient specialty care. Today, large urban polyclinics employ 15–20 categories of specialists, and polyclinics in small towns 3–5 categories. The generalist who serves for the catchment area (district doctors) is limited in the scope of services they provide. Multidisciplinary practices are built through employing new specialists, while in European countries mainly through nurses and other categories of staff. Specialists in Russian polyclinics do not supplement, but essentially replace district doctors: they accounted for 66% of visits in 2019.^1^

The scope of district doctors’ services is limited: at least 30–40% of initial visits end with referrals to a specialist or to a hospital, while in Europe only 5–15% [35, 36]. Gatekeeping is promoted, but district doctors are overloaded and not interested in expanding the scope of their services. Specialists in polyclinics have insufficient training and poorly equipped, e.g. urologists do not do ureteroscopy and ophthalmologists do not practice surgery.

**Fig. 1:**
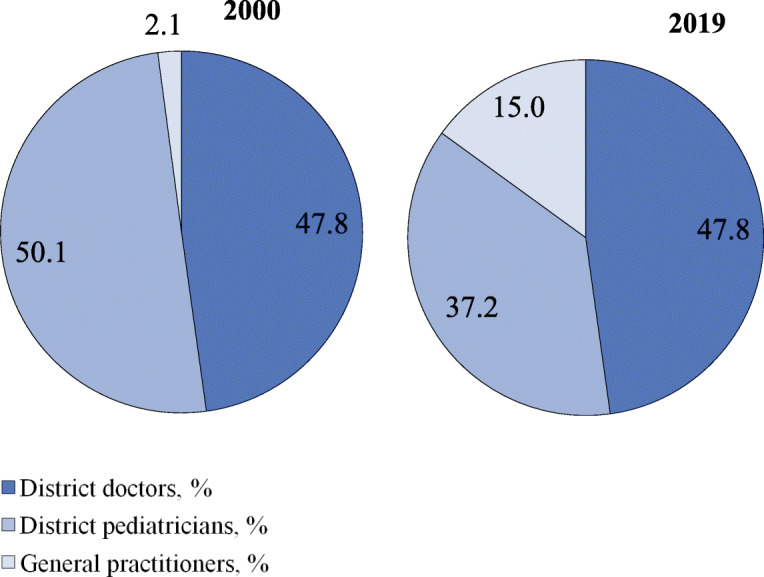
Distribution of generalists in Russia by categories in 2000, 2019. Source: Calculated from RRIH [22, 37]

Since the 1990s, some regions started replacing district doctors and pediatricians with general practitioners. But this initiative has not been supported by the federal Ministry of Health, therefore the institution of a general practitioner is not accepted throughout the country. Currently, the share of general practitioners in the total number of generalists serving a catchment area is only 15% (Fig. 1). The model of general practice is used only in some regions. The main part of the primary care in the country is provided by district doctors and pediatricians, whose task profile remains narrower than that of general practitioners. The division of primary care for children and adults is preserved. The family is not a whole object of medical care. This division is actively defended by Russian pediatricians with references to specific methods of managing child diseases.

The prevailing trend in all European HCS is to increase the role of nurses. In Russia, the participation of nurses in medical care is limited to fulfilling doctors’ prescriptions and performing ancillary functions.

### The transformation of inpatient care

#### European HCS

Due to increased costs, technological advances in diagnosis and treatment, there were changes in patterns of diseases and patients treated in hospitals. A substantial amount of inpatient care has been moved to outpatient settings with a respective decrease in bed capacity. This is an almost universal trend in European HCS [19].

Hospitals continue to be centers of high-tech care, which concentrate most difficult cases and intensify inpatient care with a corresponding decrease in the average length of stay. These changes have been promoted by the move to diagnostic related groups based payment systems and a growing integration with other sectors of service delivery.

In many European countries, most hospitals no longer act as discrete entities and have become units of hospital-physician systems which are multi-level complex adaptive structures [3]. A new function of hospital specialists is their involvement in chronic disease management in close collaboration with general practitioners, outpatient specialists, and rehabilitative and community care providers [38].

#### Russia

**Fig. 2:**
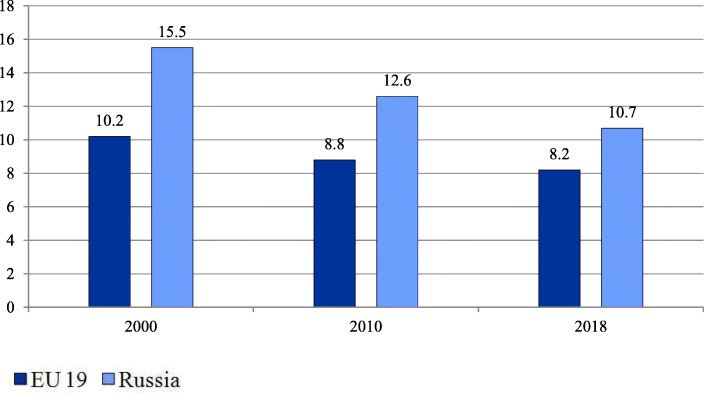
Average length of stay in hospital in EU members and Russia (days). Note: Calculated for EU 19 member states (see Methods). The EU 19 average length of hospital stay estimates are calculated as the sum of the products of inpatient care discharges by the average length of stay for each country, weighted average by the total inpatient care discharges. Source: OECD Health Statistics [18]

**Fig. 3:**
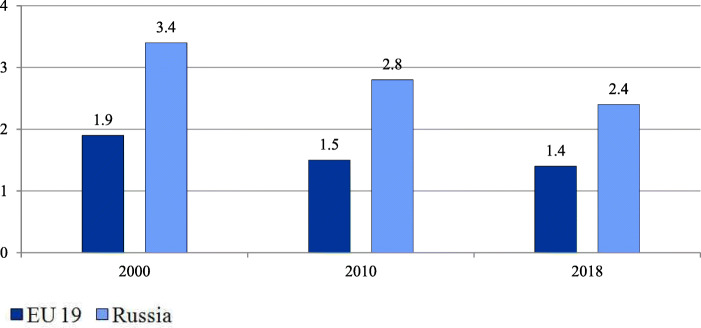
Number of bed-days per person in the EU and Russia. Note: Calculated for EU 19 member states (see Methods). EU 19 estimates are calculated as the sum of the products of inpatient care discharges by the average length of stay for each country weighted by the total population. Source: OECD Health Statistics [18]

**Fig. 4:**
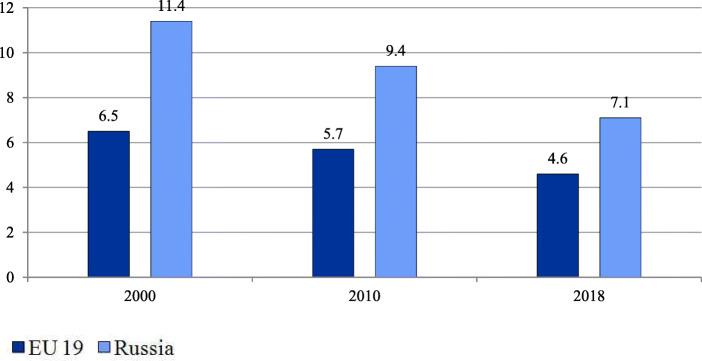
Hospital beds per 1000 people in the EU and Russia. Note: Calculated as the average for all EU 28 members weighted by the total population. Source: World Bank [20]

Over the past two decades the treatment of relatively simple cases and preoperative testing have gradually moved to day care wards and polyclinics. In annual health funding, the federal government sets decreasing targets of inpatient care which are obligatory and which regions use to plan their inpatient care. However, inpatient care discharges per 100 people have been almost stable (21.9 in 2000 and 22.4 in 2018) in contrast to the EU 19 members^2^ (18.4 in 2000 and 16.9 in 2018) [18]. The pressure of decreasing targets resulted in a drop in the average length of hospital stays (Fig. 2) and the total bed-days per person (Fig. 3). These indicators, along with bed supply (Fig. 4), decreased even faster than in the EU.

At the same time, the intensity of medical care processes in hospitals in Russia remains significantly lower than in European countries. An indicator of this is the gap in the number of hospital employees per 1000 discharged (Table 1).

**Table 1:** Number of hospital employees per 1000 inpatient discharges in EU members and Russia, 2019 (or nearest year)

Countries	Number of hospital employees per 1000 inpatient discharges
Czech Republic	79
France	107
Germany	65
Italy	95
Spain	124
United Kingdom	174
EU average*	99
Russia	50

Note: (*) - Calculated for EU 18 member states (19 countries mentioned in Methods with exclusion Finland)

Sources: Calculated for EU countries using OECD Health Statistics data [18]; data for last year available was used for number of inpatient discharges for some countries. Calculated for Russia using the data from [34].

Over the past 20 years, significant efforts have been made to deploy day wards, both in hospitals and polyclinics, to reduce the burden on hospitals. As a result, the proportion of patients treated in day wards in the total number of patients treated in hospitals increased from 7.6% in 2000 to 20.8% in 2016 [21]. However, there is fragmentary evidence that this figure is still noticeably lower than in Europe. The share of cataract surgery carried out as ambulatory cases varies in most European countries between 80 to 99% [24] but is negligible in Russia.

Despite these positive trends, the health system remains hospital centered. The number of bed-days per person remains nearly twice as high as the EU average (Fig. 3).

An important trend is the increasing concentration of hospitals. The number of hospitals halved between 2000 and 2018, mostly due to mergers, but also due to the closures of inadequately equipped hospitals. This process has led to an increase in the average size of hospitals from 156 beds in 2000 to 223 beds in 2018 [21]. This figure is higher today than in Western countries with large territories. The average hospital size in France was 130 beds in 2018 and in Germany 215 beds in 2017 [18]. In Russia, with its very low population density, the reduction in the number of small rural hospitals resulted in some accessibility problems.

At the same time, the incorporation of previously independent polyclinics into hospitals is under way. The proportion of independent polyclinics in the total number of polyclinics has decreased from 35% in 2000 to 19% in 2014 [36].

### The development of long-term care

#### European HCS

Over the last 20 years, most European countries have increasingly developed the public provision of long-term care. The number of nursing and elderly home beds per 100,000 people in the EU increased from 581.7 in 2000 to 748.3 in 2014 [19], although the pace of changes, the coverage of citizens in need of long-term care, and its organization and funding differ substantially across countries [39]. Many countries control costs by keeping people in their homes longer and shifting the responsibility for non-institutional forms of care to communities [40]. An expected outcome of investment in long-term care is the reduction of informal care utilization.

#### Russia

Compared to European HCS, long-term care is underdeveloped in Russia. The number of nursing care beds declined from 14.7 per 100,000 people in 2011 to 10.6 in 2019 [22]. The share of citizens over working age and people with disabilities receiving outpatient and inpatient care within the long-term care system in the total number of citizens over working age and people with disabilities in need of long-term care, was only 2.9% in 2019 [41].

In contrast to the European HCS, Russia has not built a strong long-term care sector with the capacity to reduce the workload of acute inpatient care settings. Hospitals have to keep some patients longer resulting in a relatively higher length of stay. Palliative care as another sector of the long-term care which started to develop only a few years ago.

### Driving forces and tools of structural changes in the Russian health care system

These changes have been driven by the federal and regional governments. They use two main tools to manage structural changes: 1) setting health care targets for the entire country and for regions, and 2) implementing vertical health care programs.

Since 1998, the federal government has annually approved a program of benefit packages for health (the Program). It sets targets for the utilization of medical care for each sector of service delivery, as well as unit cost targets. The Program is designed to balance the volumes of care with the amount of public funding. The annual versions of the Program gradually reduced the targets for inpatient care to encourage a shift to outpatient care. The federal targets are used in regional health planning. In the first decade of using the Program, the changes in the actual volume of medical care were small, but in the second decade, pressure from the federal center on the regions increased, and the gap between the federal targets and the actual utilization of care has noticeably narrowed (Table 2).

**Table 2:** The utilization of medical care in Russia: federal targets and actual values, 1998–2019

		1998	2000	2005	2010	2014	2015	2019
**Outpatient care**								
Number of physician visits per person	Target	9.198	9.198	9.198	9.5			
	*Actual*			*8.523*	*9.312*			
Number of physician visits for preventive services per person	Target					2.77	2.9	3.61
	*Actual*					*3.772*	*3.735*	*3.598*
Number of outpatient care episodes per person (normalized number of visits per episode)	Target					2.12	2.15	2.24
	*Actual*					*2.019*	*1.915*	*1.617*
**Day care**								
Patient-days per person	Target	0.749	0.749	0.577	0.590	0.665	0.675	
	*Actual*			*0.457*	*0.523*	*0.597*	*0.619*	
**Inpatient care**								
Bed-days per person	Target	2.902	2.813	2.813	2.78			
	*Actual*	*3.317*	*3.298*	*3.038*	*2.733*			
Hospitalization per person	Target					0.197	0.193	0.189
	*Actual*					*0.205*	*0.200*	*0.192*
**Ambulance (Emergency) care**								
Emergency calls per person	Target	0.340	0.318	0.318	0.318	0.318	0.318	0.300
	*Actual*	*0.346*	*0.362*	*0.339*	*0.336*	*0.303*	*0.307*	*0.293*

Note: The target indicators have changed several times, which does not allow the collection of long time series. Table 1 is built using the target indicators set in the corresponding period of time

Sources: For target indicators – Government of the Russian Federation [42]; for actual indicators – Ministry of Health of the Russian Federation [43].

The development of the legislation on the delimitation of responsibility between levels of government, carried out in the last two decades, has consistently strengthened the regional governments role in restructuring medical care delivery. In 2012, almost all resources of health care governance were transferred from the municipal to the regional level (including the governance of primary health care. During the period 2000–2019 the number of public hospitals has decreased by 2.2 times, the number of hospital beds by 1.5 times, polyclinics 1.3 times, feldsher-obstetric posts 1.3 times.^3^

When oil prices increased, the federal government poured additional resources into vertical programs. They are administered by the federal Ministry of Health and regional governments. The major programs: the ‘Priority national health project’ (2004–2012), the Prophylactic Program (2008 – ongoing), and regional programs for the modernization of health care (2011–2013). All additional and some basic resources are earmarked in an attempt to develop the highest priority activities: preventive care, obstetric care, cardiovascular surgery, oncology, etc.

The role of the centralized administration of these priority programs is controversial. The federal government initiated them, provided regions with additional funding, and made the program’s targets a priority of health policy. According to interviews with federal and regional officials, the implementation of programs is heavily controlled by the federal government: practically all decisions on specific activities, target indicators and resource allocation are approved on the federal level. The Russian regions have low flexibility to respond to local needs such as variation in disease incidence, the capacity of health care, or vulnerable population groups.

Structural changes in the provision of inpatient care were prompted by the introduction of a diagnostic related groups based payment system in the early 2010s. This was initiated by the federal government and implemented with the participation of the World Bank experts. It makes more profitable for hospitals to reduce the duration of hospitalizations and to complicate the structure of inpatient treatment [44].

## Discussion

We found that despite significant differences in health care organization, some structural changes in Russia have followed the general European trends. A similar rise in the coverage of the population with screenings is underway in Russia. There is a clear tendency to replace some inpatient care with day care. The volume of inpatient care is reducing —mostly due to a significant decrease in the length of stays, while the rate of hospital admission remains relatively stable. As in the most European HCS, the concentration of medical organizations and the formation of large outpatient and inpatient complexes is developing.

However, there are some substantial differences: the development of prevention programs is relatively less focused on the most vulnerable target groups and on local needs; primary care specialization is much stronger than in European HCS; the role of first contact generalists is waning; the worldwide tendency of increasing the role of nurses is almost invisible in Russia; long-term care is starting to develop but is still at a very low level and palliative care is in its infancy; integration in the health system are much less pronounced—both at the level of individual medical organizations and between health sectors.

The reasons for these differences are rooted in the specific features of health governance in Russia.

The Semashko model, by virtue of its genesis, reproduces the state administration patterns of a planned economy. The main driving force of changes is the bureaucracy. Its managerial activities are guided by the mechanism described by J. Kornai: ‘postponement, putting out the fire, postponement’ [45]. The governance focuses on mobilizing and distributing available resources to solve or mitigate the most pressing problems - ‘fire fighting’. This is what determines the fragmentation of structural changes in Russian health care compared to structural changes in European countries.

Materials of interviews with heads of federal and regional health authorities suggest that in the existing governance system each of its levels must demonstrate the success of its activity exclusively to the higher levels of management. It is easier to achieve success when solving problems of optimizing the volume of medical care and the organizational structure of medical institutions, and much more difficult when solving problems of improving the efficiency of all elements of medical care system, which requires changes in their functionality and ways of interaction. It requires more financial resources and better management at all levels of health governance.

A number of deeply rooted limitations for carrying out structural transformations in Russian health care can be highlighted.

Firstly, the low capacity of primary care providers and to some extent the unwillingness of patients to replace inpatient care with outpatient treatment prevents a shift of patients from hospitals to polyclinics.

Secondly, a feature of the Russian health care system is the weak development of horizontal links between medical organizations related to different levels of medical care, and between medical workers within medical organizations working in different departments [36, 46, 47]. The interaction of different providers is carried out mostly through vertical channels. This is a serious obstacle to the development of horizontal integration [36].

Thirdly, democratic institutions for the development of health care are historically underdeveloped in Russia and this influences the choice of health policy priorities. According to interviews with heads of regional health authorities, the role of local communities is negligible, and the role of the medical community is marginal. Professional organizations are rarely involved in decision-making on health issues. The input of public councils to government bodies is largely imitative. Information about the activities of the system as a whole and of individual medical organizations is restricted for public use. This enables health authorities to focus on achievements in their reports, while hiding shortcomings. Feedback from patients, and society as a whole, is poorly expressed.

## Conclusions

Russian health care, whose genetic basis was the Soviet Semashko model, after a difficult ‘survival’ period in the 1990s, underwent significant structural changes over the next two decades. To a large extent, the directions of these changes have coincided with European trends. The prevention and the early detection of diseases have developed intensively. The reduction in hospital bed capacity and inpatient care was accompanied by an intensification of inpatient treatment and a decrease in the average length of stay. Russia has followed the European trend of service delivery concentration in hospital-physician complexes, while the increase in the average size of hospitals is even more substantial. Structural changes in primary care are much less pronounced. The resources and competences of providers and the governance of primary care are still not enough to abolish the hospital-centered model of service delivery. Russia has intensively implemented vertical health care programs to develop the priority activities, but still significantly lags in the level of development of horizontal ties among services providers.

Specific structural changes in Russia are rooted in the organization and governance of service delivery. The interests of federal and regional bureaucracies, which act as the main drivers of changes, are pushing them to prioritize the changes in volumes of medical care and organizational structure of health care providers and not spend a lot of effort on improving their functionality and modes of interaction between providers of medical care. An important role is also played by the low capacity of primary care units to provide quality care.

To respond effectively to modern global challenges, reduce mortality, and improve the health of the population, structural transformations in Russian health care must be intensified with the focus on strengthening primary care, the further integration of care, and an accelerated development of new structures that mitigate the dependence on inpatient care.

## Data Availability

The data used and analysed during the current study are publicly available.

## Abbreviations

EU: European Union
HCS: Health Care System
OECD: Organization for Economic Co-operation and Development
WHO: World Health Organization

## Footnotes

1 Calculated using data from [34].

2 See Methods.

3 Calculated using data from [21].
